# Precision medicine approaches to lung adenocarcinoma with concomitant *MET* and *HER2* amplification

**DOI:** 10.1186/s12885-017-3525-9

**Published:** 2017-08-10

**Authors:** Doo-Yi Oh, Kyungsoo Jung, Ji-Young Song, Seokhwi Kim, Sang Shin, Yong-Jun Kwon, Ensel Oh, Woong-Yang Park, Sang Yong Song, Yoon-La Choi

**Affiliations:** 1 Department of Pathology and Translational Genomics, Samsung Medical Center, Sungkyunkwan University School of Medicine, 81 Irwon-ro, Gangnam-gu, Seoul, 06351 South Korea; 2 Institute for Refractory Cancer Research, Samsung Medical Center, Seoul, South Korea; 3 Department of Health Sciences and Technology, SAIHST, Sungkyunkwan University, Seoul, South Korea; 4 Samsung Genome Institute, Samsung Medical Center, Seoul, South Korea

**Keywords:** Met, HER2, Patient-derived cells, Lung cancer

## Abstract

**Background:**

Patient-derived xenograft (PDX) models are important tools in precision medicine and for the development of targeted therapies to treat cancer patients. This study aimed to evaluate our precision medicine strategy that integrates genomic profiling and preclinical drug-screening platforms, in order to personalize cancer treatments using PDX models.

**Methods:**

We performed array-comparative genomic hybridization, microarray, and targeted next-generation sequencing analyses, in order to determine the oncogenic driver mutations. PDX cells were obtained from PDXs and subsequently screened in vitro with 17 targeted agents.

**Results:**

PDX tumors recapitulated the histopathologic and genetic features of the patient tumors. Among the samples from lung cancer patients that were molecularly-profiled, copy number analysis identified unique focal *MET* amplification in one sample, 033 T, without RTK/RAS/RAF oncogene mutations. Although *HER2* amplification in 033 T was not detected in the cancer panel, the selection of *HER2*-amplified clones was found in PDXs and PDX cells. Additionally, MET and HER2 overexpression were found in patient tumors, PDXs, and PDX cells. Crizotinib or EGFR tyrosine kinase inhibitor treatments significantly inhibited cell growth and impaired tumor sphere formation in 033 T PDX cells.

**Conclusions:**

We established PDX cell models using surgical samples from lung cancer patients, and investigated their preclinical and clinical implications for personalized targeted therapy. Additionally, we suggest that MET and EGFR inhibitor-based therapy can be used to treat MET and HER2-overexpressing lung cancers, without receptor tyrosine kinase /RAS/RAF pathway alterations.

**Electronic supplementary material:**

The online version of this article (doi:10.1186/s12885-017-3525-9) contains supplementary material, which is available to authorized users.

## Background

Recent advancements in time and cost reductions of next-generation sequencing technologies, allow for the characterization of the cancer genome in a period when treatment decisions are made, offering the opportunity to increase the therapeutic efficacy by targeting oncogenic driver alterations [1]. However, it is difficult to integrate genomic profiling with the decision-making process of cancer treatment, as the interpretation of genomic data is still challenging.

Lung cancer is the most common cause of cancer-related mortality, in both men and women worldwide [2]. Non-small-cell lung cancer (NSCLCs) is the most prevalent lung cancer type, and it can be further subcategorized using patient gene mutation profiles. Personalized treatment strategies can be developed based on the oncogenic driver alterations that can be targeted with tyrosine kinase inhibitors (TKIs). The oncogenic driver alterations identified in NSCLCs are *epidermal growth factor receptor* (*EGFR*), *KRAS*, *BRAF*, AKT1, *HER2*, *MEK1*, *MET*, *NRAS*, *PIK3CA* mutations or translocations in *anaplastic lymphoma kinase* (*ALK*), *RET*, and ROS1 genes [3]. However, most NSCLCs lack identifiable oncogenic driver alterations, and these patients are still treated with conventional chemotherapy.

Despite the successful development of EGFR- or *EML4-ALK*-targeted TKIs, treatment options remain limited for patients with advanced lung cancer lacking an identifiable oncogenic driver alteration. Several other molecular markers that are altered in NSCLCs have been identified, such as phosphatidylinositol 3-kinase (PI3K) (2% of NSCLCs) [4], AKT, HER2 (2% of NSCLCs), and MET [3]. Tyrosine kinases MET and HER2 are described as novel promising therapeutic targets.

*MET* occurs in 5% of NSCLCs [5], and the *MET* mutations have been detected in 10% of NSCLCs [6]. Aberrant MET pathway activation has been identified as an important oncogene addiction mechanism in different solid tumors and it seems to correlate with poor clinical outcomes and metastatic progression. MET dysregulation can lead to an acquired resistance to EGFR-TKIs therapy [7] or it can be activated, de novo*,* in the absence of any other driver mutation [3], suggesting that this protein is a potential key target for molecular therapies. *HER2* amplifications and mutations have been detected in 10–20% and 2–4% of NSCLCs [8]. *HER2* mutations in NSCLC confer relative resistance to conventional chemotherapy [9], and *HER2* amplification is one of the mechanisms of acquired resistance to EGFR TKIs, suggesting that HER2 targeted therapy can be considered as a therapeutic option in NSCLC patients with such HER2 alterations.

Recent Genomic Identification of Significant Targets in Cancer studies provided the evidence of *MET* and *HER2* amplifıcations in NSCLCs lacking identifiable oncogenic driver alterations, warranting detailed investigations of the effects of MET and HER2 inhibitors [3]. Patients with de novo *MET* amplifications showed the reduced response to the treatment with receptor tyrosine kinases TKI [10], while ALK negative squamous cell lung cancer patients with de novo *MET* amplifications showed major partial response to a dual MET/ALK inhibitor [11]. HER2 overexpression, as assessed by immunohistochemistry (IHC), is found in 2–6% of NSCLC cases [12], but the sensitivity to trastuzumab has not been studied in detail in this patient population. Unlike *EGFR*, *RAS*, *ALK*, and *ROS1* genes, which are known to play important roles in tumorigenesis, the role of MET and HER2 as oncogenic driver genes, remain to be confirmed.

We have established lung cancer patient-derived xenograft (PDX) models, in order to investigate new therapeutic strategies using preclinical drug-screening platforms. Additionally, following the analyses of the patient-derived tumors and PDXs, we found a MET and HER2-overexpressing tumor, without receptor tyrosine kinase (RTK)/RAS/RAF oncogenic alterations. Histopathological and genomic characterization of the investigated tumors, and in vitro drug screening using PDX cells, were performed. The results obtained suggest that MET and EGFR inhibitor-based therapies can be investigated using a preclinical platform, which accurately mimics the clinical situation of lung cancer patients without RTK/RAS/RAF oncogene alterations.

## Methods

### Patient tissue samples

This study and all the experimental procedures were approved by the Samsung Medical Center (Seoul, Korea) Institutional Review Board, and written informed consents were obtained from all participants (No. 2010–04-004). Tumors were classified as NSCLCs, based on the World Health Organization (WHO) criteria. Patients were categorized as never-smokers (<100 cigarettes in their lifetime) or ever-smokers (≥100 cigarettes in their lifetime), according to their smoking status. NSCLC histologic subtypes and stages were classified according to the WHO criteria [13] and the American Joint Committee on Cancer staging system [14], respectively. Surgical specimens were divided into three parts for implantation into immunodeficient mice, DNA/RNA extraction, and pathologic assessment, within6 hours after surgery.

Non-malignant normal lung tissue samples were taken from the far margins of the lung resections, which were grossly and microscopically negative for tumor tissue. Lung tissue samples were minced with scalpels into 1 mm pieces, and afterward enzymatically disaggregated to create single-cell suspensions, by incubating them with 1 mg/mL collagenase P (Roche Genentech, San Francisco, CA, USA) and 0.1 mg/mL DNase I (Applied Biosystems, Foster City, CA, USA) in RPMI 1640 medium with 10% fetal calf serum (FCS) for 16 h, with constant stirring. Each well of a 6-well culture plate (Corning, NY, USA) was inoculated with 100 × 10^3^ viable cells in 4 mL RPMI 1640 medium with 10% FCS.

### Cell lines

The H1975 (*EGFR* mutation; L858R/T790 M; CRL-5908™, American Type Culture Collection (ATCC) University Boulevard Manassas, VA, USA) and H2228 (*EML4-ALK*; CRL-5935™, ATCC University Boulevard Manassas, VA, USA) cell lines were obtained from the American Type Culture Collection (ATCC University Boulevard Manassas, VA, USA), and maintained in RPMI 1640 medium supplemented with 10% fetal bovine serum (FBS; 16,000–044; Invitrogen, Grand Island, NY, USA) at 37 °C in 5% CO_2_. All of the cell lines used were authenticated by short tandem repeat (STR) profiling before a new series of experiments was begun and were kept in culture for no more than 3 months.

### PDX establishment

Athymic nude and NOG (IL2 receptor g-chain truncated NOD/SCID) mice were utilized (Orient Bio, Sungnam, Korea). Prior to the transplantation, 6- to 8-week old female mice were anesthetized with CO_2_. Primary NSCLC samples, minced into fragments of approximately 1 mm^3^, in high-concentration Matrigel Basement Membrane Matrix (BD Biosciences, San Jose, CA, USA), were directly implanted subcutaneously (*n* = 4–5 for each tumor sample). Mice were maintained and the development of xenograft tumors was observed for 6 months. For subcutaneous implantation, xenograft tumor engraftment was defined as an appearance of a palpable mass of >1 mm in diameter, which was pathologically confirmed. For intracranial injections, body weight loss of >20% and pathologic confirmation were considered as a successful PDX model establishment. The origin of each xenograft was validated by short tandem repeat DNA fingerprinting. PDX tumors were harvested and divided into three parts for the generation of the second in vivo passage xenograft tumors, DNA/RNA extraction, and histopathologic examination. This study was approved by the Institutional Animal Care and Use Committee of the Samsung Biomedical Research Institute (protocol No. H-A9–003) and carried out the guidelines approved by the Institute of Laboratory Animal Resources Guide.

### Histologic examination and IHC

Hematoxylin and eosin (H&E) staining was performed on all paraffin blocks with the tissue samples obtained from both patients and PDXs. The PATHWAY® anti-MET (sp44; Ventana Medical Systems, Tucson, AZ, USA) and anti-HER2/neu (4B5; Roche, Basel, Switzerland) antibodies were used for MET and HER2 immunohistochemical staining. The sections (3 mm) were deparaffinized, rehydrated, and antigen retrieval was performed in citrate buffer (pH 6.1) at 95 °C for 40 min. Diaminobenzidine was used as the chromogen. The sections were counterstained with hematoxylin. The BenchMark XT IHC/ISH staining instrument (Ventana Medical Systems, Tucson, AZ, USA) was utilized. MET and HER2-expressing lung and breast cancer tissues were used as positive controls.

### Array comparative genomic hybridization (aCGH)

The aCGH was detected genetic variations, including deletions and duplications using the Agilent Human Whole Genome CGH 8 × 60 K microarray (Agilent Technologies, Santa Clara, CA). Test and reference DNA samples were labeled by random priming with either Cy3-dUTP or Cy5-dUTP using the Agilent Genomic DNA Labeling Kit PLUS (Agilent Technologies). All slides were scanned on an Agilent DNA microarray scanner. Data were obtained using the Agilent Feature Extraction Software 9 (Agilent Technologies) and analyzed the ADM-2 statistical algorithms with 6.0 sensitivity thresholds as described previously [15].

### Genetic alterations analysis using the cancer panel

The Cancer Panel is a targeted next-generation sequencing assay that was developed, validated, and provided by the Samsung Genome Institute (Samsung Medical Center, Seoul, Korea). It includes all exons from 381 cancer-related genes and 31 introns from five genes recurrently rearranged in cancer. Using the Illumina HiSeq 2500 (San Diego, CA, USA), the captured libraries underwent paired-end high-depth sequencing (target >800× coverage). Data were analyzed using an automated bioinformatics pipeline, designed to detect various genetic alterations, including single nucleotide variations, insertions and deletions, gene amplifications and deletions, and gene fusions.

### Quantitative polymerase chain reaction (qPCR)

Genomic DNA was isolated from FFPE tumor specimens and *MET* and *HER2* copy number amplification was performed by a PRISM 7900HT Fast Realtime PCR system (Applied Biosystems). All quantitative PCR reactions were performed in triplicate using the SYBR Green method. The PCR conditions were: preheating at 50 °C for 2 min; 95 °C for 10 min; 40 cycles at 95 °C for 15 s and 60 °C for 1 min. *MET* and *HER2* copy numbers were calculated by comparison to *ALB*, located at 4q11-q13, and normalized to normal tissue genomic DNA. Primers for *MET* and *HER2* copy number analysis were the following: *MET* forward, 5′-ATTGGTGATTGCTTGGGTAGTT-3′; *MET* reverse, 5′-CCTGTGGGTTTACTTTGGTTG-3′; *HER2* forward, 5′-GGAGGATGTGCGGCTCG-3′; *HER2* reverse, 5′-CATGGTTGGGACTCTTGACCA-3′; *ALB* forward, 5′-TGAAACATACGTTCCCAAAGAGTTT-3′; *ALB* reverse, 5′-CTCTCCTTCTCAGAAAGTGTGCATAT-3′. All PCR products were purified using a PCR purification kit and directly sequenced by standard procedures using forward and reverse primers.

### In situ hybridization

Sequential in situ hybridization procedures for *MET*, *HER2*, *CEN7*, and *CEN17* signal detection were conducted using INFORM *MET* and *HER2* DNA, and *CEN7* and *CEN17* probes (Ventana Medical Systems, Tucson, AZ, USA). Fluorescence in situ hybridization was used dual-color probes to *MET* and *CEP7* from PathVysion™ (LSI® *MET* Spectrum Orange™ and *CEP7* Spectrum Green™; Abbott, San Francisco, CA, USA).

### Microarray experiments

Four patients (designated as 033 T and 694 T, and PDXs 033 T and 694 T) were investigated, and a complete case set consisted of a primary tumor sample and corresponding xenografts. Total RNA was extracted from the patient tissues and xenografts containing >80% tumor cell content. Extracted RNA was hybridized to Agilent 60 K expression microarrays, according to the manufacturer’s instructions.

### Primary in vitro short-term culture

Xenograft tumor specimens were dissociated into single cells according to previously published protocol [16]. Dissociated cells were cultured in Neurobasal medium-A, supplemented with N2 (×1/2; Life Technologies, Carlsbad, CA, USA), B27 (×1/2; GIBCO), basic fibroblast growth factor (bFGF; 25 ng/mL; R&D Systems, Minneapolis, MN, USA), EGF (25 ng/mL; R&D Systems, Minneapolis, MN, USA), neuregulin 1 (NRG; 10 ng/mL; R&D Systems, Minneapolis, MN, USA), and insulin-like growth factor 1 (IGF1; 100 ng/mL; R&D Systems, Minneapolis, MN, USA). Growth factors were supplemented every 3 days. When spheres appeared in the suspension culture, they were dissociated using StemPro Accutase (Life Technologies, Carlsbad, CA, USA), and reseeded in the suspension culture medium. Cells were grown in complete medium and treated with inhibitors 1 day after seeding. Five days later, the surviving cells were quantified by WST-1 staining (Roche Genentech, San Francisco, CA, USA).

### In vitro drug sensitivity assay

Primary cultures of PDX cells in the serum-free sphere culture conditions were seeded in 384-well plates, at 500 cells per well. Two hours after plating, cells were treated with a drug library in three-fold and 10-point serial dilutions (*n* = 3, for each condition). Cells were incubated for 6 days at 37 °C, in a 5% CO_2_ humidified incubator, and the cell viability was analyzed using an adenosine triphosphate monitoring system based on firefly luciferase (ATPlite 1step; PerkinElmer, Waltham, MA, USA). The drug library was composed of 17 targeted agents that were included in the clinical guidelines or current clinical trials for the treatment of NSCLC (gefitinib, erlotinib, lapatinib, afatinib, neratinib, caneratinib, dacomitinib, infigratinib, vandetanib, sunitinib, imatinib, CI-1040, vemurafenib, palbociclib, crizotinib, sonidegib, buparlisib; all purchased from Selleckchem, Houston, TX, USA). The drugs were stored and diluted according to the manufacturer’s instructions. The tested concentrations for each drug were empirically derived, in order to investigate a clinically relevant spectrum of drug activities. IC_50_ values were calculated as an average of triplicate experiments using S+ Chip Analyzer (Samsung Electro-Mechanics Company, Ltd., Suwon, Korea). In order to investigate the effects of the treatment with targeted agents, signal transduction assays were performed using primarily cultured NSCLC PDX cells, which were maintained overnight in serum-free sphere culture conditions, without growth factors, incubated for 1 h with each inhibitor, and pulsed with original culture medium supplemented with bFGF/EGF/NRG/IGF for 15 min.

### Western blotting

Immunoblotting was performed with following antibodies: MET (Santa Cruz, sc-161), phospho-MET (Tyr1234/1235; Cell Signaling Technology, 3077), p42/44 MAPK (Cell Signaling Technology, 9102), phospho-p42/44 MAPK (Thr202/Tyr204; Cell Signaling Technology, 4370), phospho-AKT (Ser473; Cell Signaling Technology, 9271), AKT (Cell Signaling Technology, 9272), GAPDH (Santa Cruz, sc-25,778), STAT3 (Cell Signaling Technology, 9139), phospho-STAT3 (Tyr705; Cell Signaling Technology, 9145), HER2 (Cell Signaling Technology, 2165), anti-HER2 (phospho Y877; Abcam, ab108371).

### Statistical analysis

The Mann-Whitney U test was used to evaluate the differences between groups in both in vitro and in vivo assays. All statistical tests were two-sided, and *P* values <0.05 were considered statistically significant. SPSS software (version 17.0; SPSS, Chicago, USA) was used for all statistical analyses.

## Results

### Identification of aberrant MET and HER2 expressions in NSCLCs

**Fig. 1:**
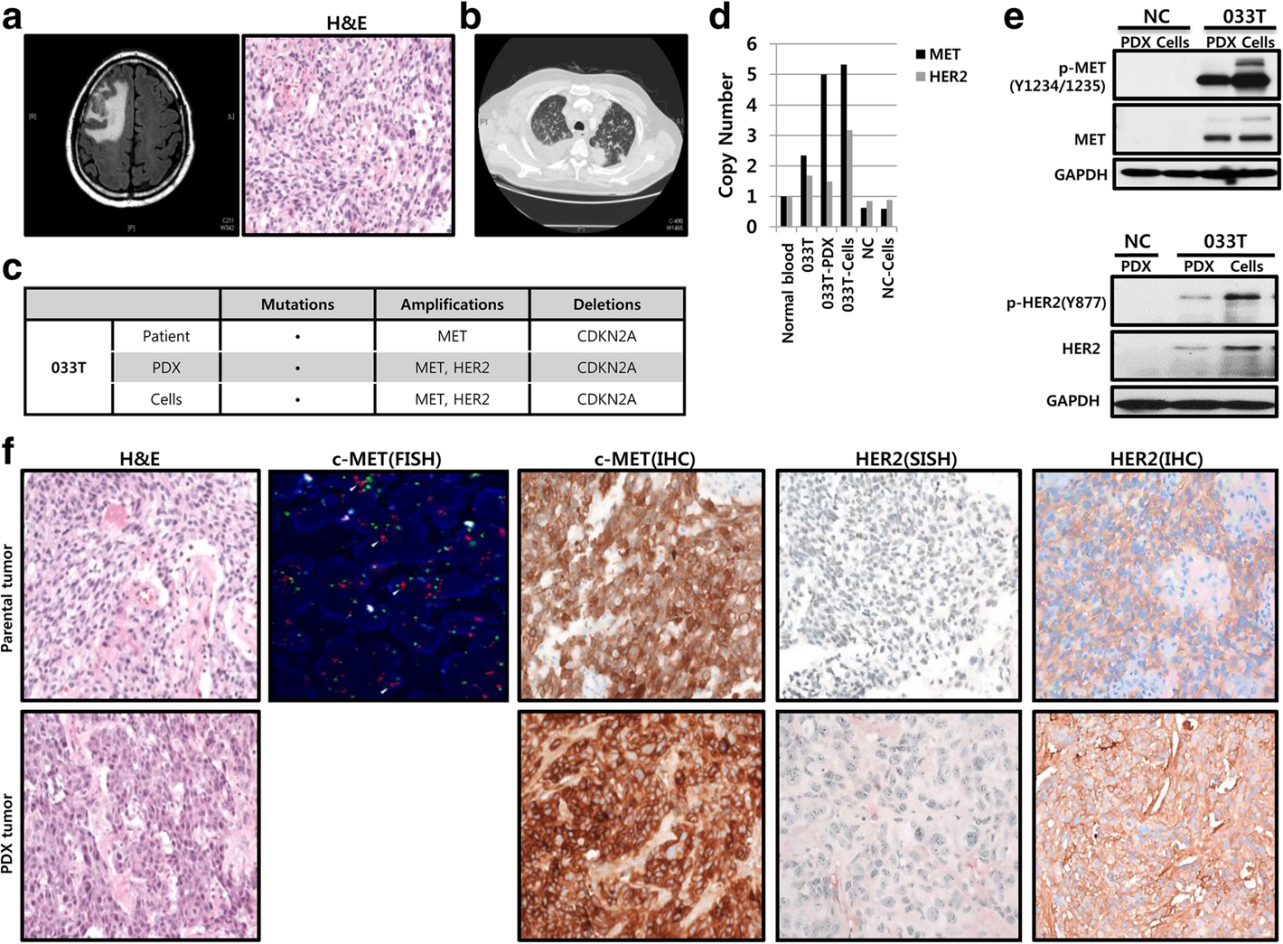
MET and HER2 expression levels in 033 T tumor sample. **a** Brain MRI showed a relatively well-demarcated enhancing mass in right frontal lobe. **b** Chest CT showed multiple masses, measuring up to 4 cm, in 033 T patient. **c** Genomic profiling of focal amplifications in 033 T patient derived tumor, PDXs, and PDX cells identified focal gains of *MET* and *HER2.* **d** Quantitative polymerase chain reaction gene copy number analysis was performed in order to detect *MET* and *HER2* amplifications. NC; patient 694 T samples, used as negative controls, did not show *MET* and *HER2* amplification. **e** MET, HER2, phosphorylated MET, phosphorylated HER2, and GAPDH as a loading control were analyzed using western blot. **f** Histologic comparison between the patient tumor sample and PDX tumor. The representative areas of each patient tumor sample and the corresponding PDXs were stained with H&E, MET, and HER2 antibodies. Fluorescence in situ hybridization of *MET* revealed amplification in many tumor cells (*red dots*). Silver in situ hybridization of *HER2* showed amplification of *HER2* in the patient tumor sample and PDX tumor (*black dots*). MET and HER2 immunohistochemical staining showed distinctive expression patterns in the patient-derived sample and PDX tumor sample (MET, HER2, ×200)

One lung cancer patient (033 T) visited the hospital complaining of weakness in the left arm and leg. Brain MRI was performed, and it revealed a 2.5 cm big mass in the frontal lobe. He was admitted to the neurosurgery department, and the observed lesion in his brain was resected. Histologic examination revealed that the well-circumscribed mass consisted of poorly differentiated round to ovoid cells, with hyperchromatic nuclei and high nuclear-to-cytoplasmic ratio. TTF-1 immunohistochemical staining was positive for the tumor cells, and the mass was diagnosed as poorly differentiated metastatic adenocarcinoma (Fig. 1a). Additional search for a primary malignant lesion was performed. Chest CT revealed multiple masses measuring up to 4 cm in the lung parenchyma, with multiple regional lymphadenopathy (Fig. 1b). The patient received palliative chemotherapy based on Alimta (pemetrexed) and cisplatin, and the whole-body radiotherapy. The treatment was unsuccessful and the patient died 1 year later.

**Fig. 2:**
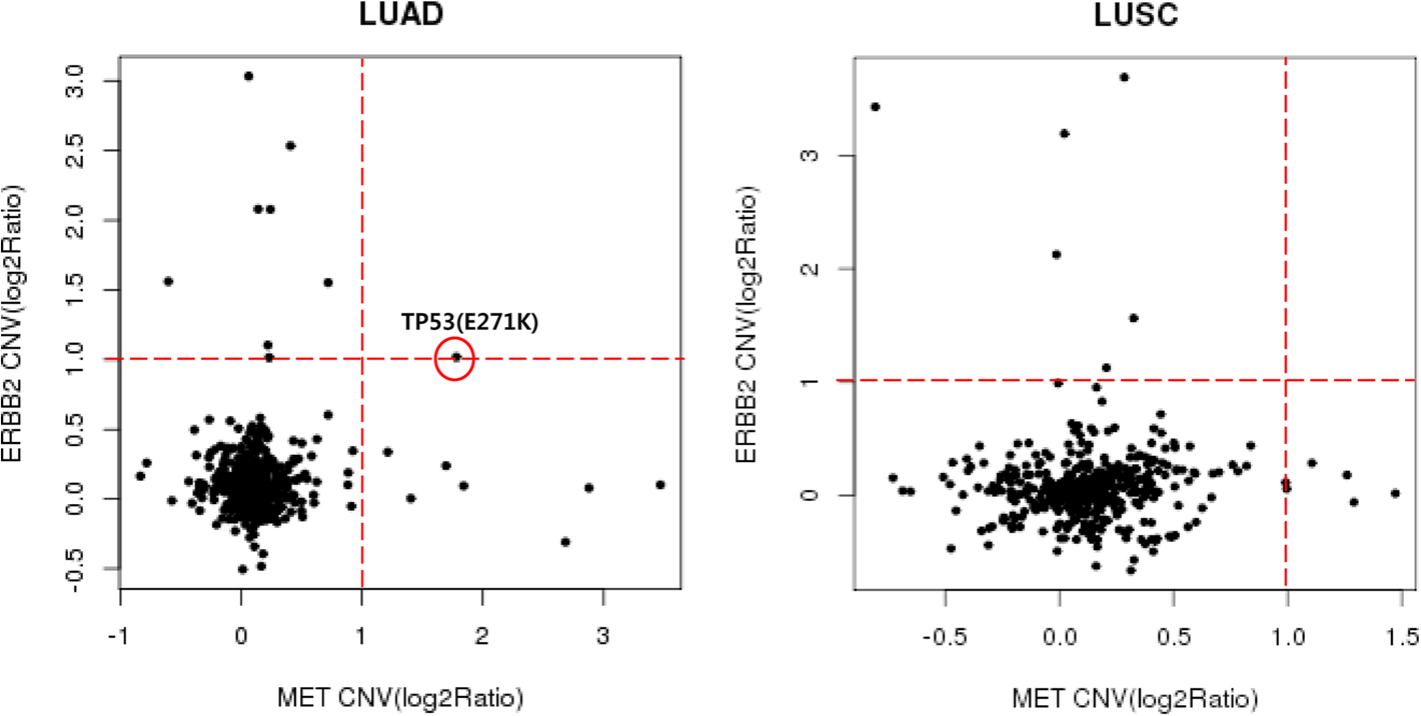
Identification of *MET* and *HER2* amplifications in lung cancers. The analysis of focal amplifications in lung adenocarcinoma (LUAD; *n* = 494) and lung squamous cell carcinoma (LUSC; *n* = 492). The Cancer Genomic Atlas samples identify focal gains of *MET* and *HER2* that are specific to the LUAD

We analyzed single nucleotide variations, copy number variations, and translocations in this tumor sample using the Cancer Panel. We did not found significant targetable oncogenic mutations in the sample when we analyzed genes that are reported in the COSMIC database (available at: http://cancer.sanger.ac.uk/cancergenome/projects/cosmic). The Cancer Panel identified *MET* amplification and *CDKN2A* deletion in 033 T sample and *HER2* was amplified in the PDXs and PDX cells but not in the patient tumor sample (Fig. 1c). We analyzed The Cancer Genomic Atlas database to find out the samples with *MET* and *HER2* amplifications of lung cancer. 1 of the 494 available lung adenocarcinoma samples exhibited *MET* and *HER2* amplifications without a known RTK/RAS/RAF activating mutation (Fig. 2). In order to confirm whether *MET* and *HER2* were amplified in the patient tumors, PDXs, and PDX cells, we analyzed MET and HER2 expressions using qPCR and microarray. Patient 694 T samples, used as negative controls, did not show *MET* and *HER2* amplification, while the 033 T tumor samples were positive for MET and HER2 expressions, and both the PDXs and PDX cells had very stable MET and HER2 expression levels (Fig. 1d). MET and HER2 expressions were detected by western blotting in 033 T but not in negative control samples (Fig. 1e). Furthermore, we investigated MET and HER2 expressions in 033 T samples following fluorescence in situ hybridization, silver in situ hybridization, and immunohistochemical staining (Fig. 1f). *MET* and *HER2* amplifications in the patient tumor samples and PDXs were demonstrated by fluorescence in situ hybridization and silver in situ hybridization as well (Fig. 1f). Microscopic examination of patient tumors and corresponding PDXs revealed that morphological characteristics (cell and tissue architecture) were retained. Although the tumor histology examination showed little difference between the tumor samples and PDXs following H&E staining (Fig. 1f, left panel), HER2 immunohistochemical staining pattern and intensity revealed a dramatic difference. This tumor sample demonstrated only cytoplasmic HER2 staining, showing immunopositivity for 60% of the total tumor volume (intensity: strong, 0%; moderate, 5%; weak, 60%; Fig. 1f). However, in PDX tumor, 60% of the tumor cells had positive membranous HER2 (strong intensity), and the remaining 40% showed weak or moderate cytoplasmic HER2.

### PDXs and PDX cells retain transcriptional and morphological characteristics of the patient-derived tumors

We investigated whether the genetic features of the patient-derived tumors were recapitulated by PDX counterparts, using aCGH. Both PDXs and PDX cells and the patient-derived tumors exhibited conserved aCGH profiles (Additional file 1: Figure S1). Gene expression analyses were performed using the patient-derived tumors and corresponding PDXs, for 15 common genes whose products may be involved in key pathway components for RTK signaling, proliferation, translation, and cell cycle progression. The core level gene expression analysis was used to build heat maps, which show that there is no significant difference in the expression of any genes between PDXs and matched PDX cells, suggesting that PDXs and the paired PDX cells have similar genomic profiles (Additional file 2: Figure S2). Furthermore, we investigated whether the genomic features of the patient-derived tumors were retained by their PDX counterparts, using STR analysis. STR profiles of 033 T tumor samples, and PDXs and PDX cells were identical to the original tumor profile at all STR loci [16]. We performed a quality control testing for PDXs and PDX cells, in order to determine the accuracy of PDX genomic statuses, and the absence of cross-contamination. The mean Ct values for the human *ALB* were much lower than for the mouse *ALB*, indicating that human DNA was much more abundant than mouse DNA in the PDX tumor samples, and mouse *ALB* was not detected in 033 T PDX cells (data not shown).

### A useful model for the evidence-based precision medicine

**Fig. 3:**
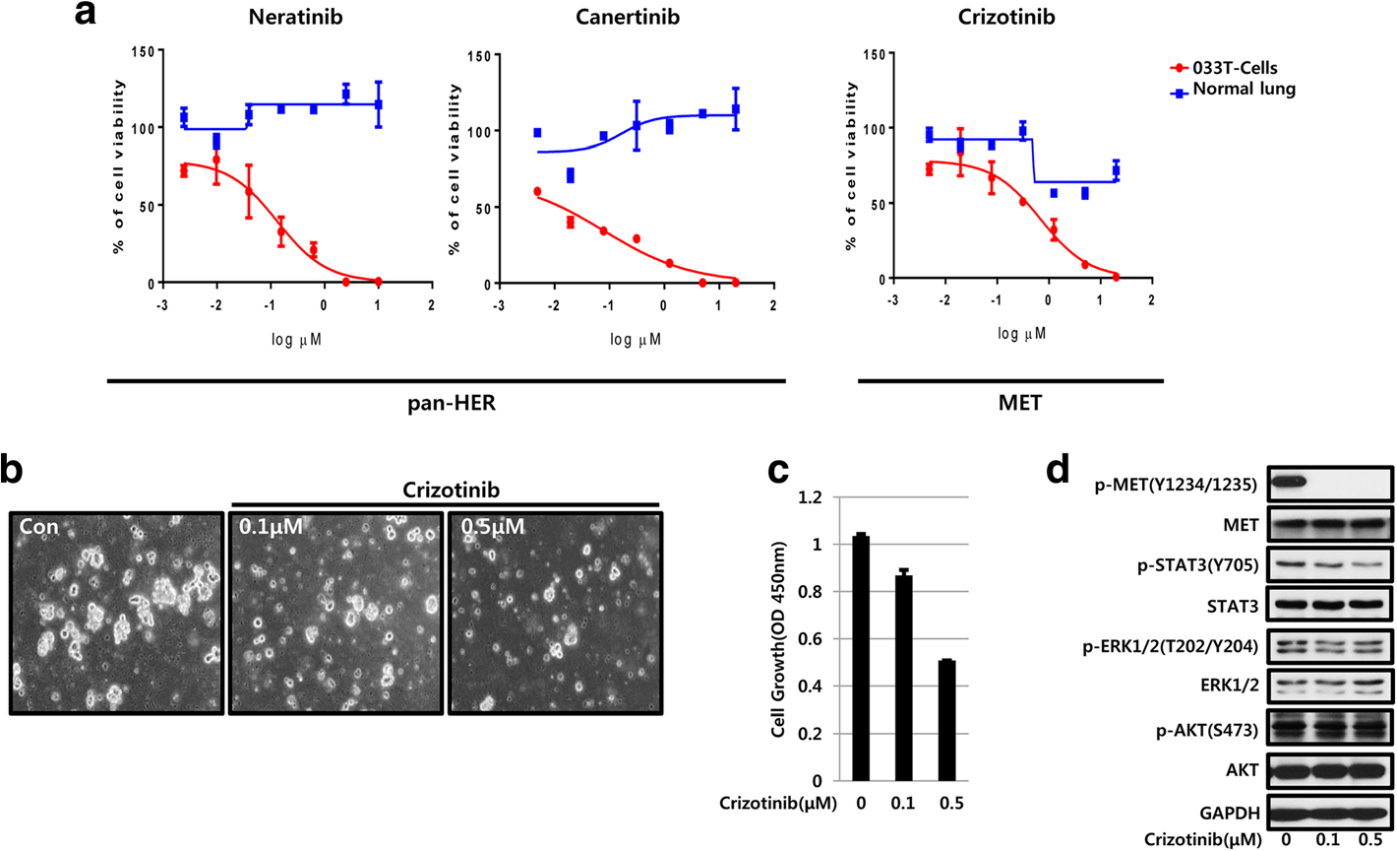
Targeted therapy using 033 T PDX cells. **a** In vitro sensitivity of primary PDX cells (033 T) and normal lung tissues to a panel of targeted therapeutic agents. Primary PDX cells were cultured from the established xenograft tumors in serum-free sphere culture conditions. Dose response curves for the 3 molecular targeted agents, such as neratinib, canertinib, and crizotinib. **b** Treatment with MET inhibitor, crizotinib impaired tumor sphere formation in 033 T cell cultures. **c** Treatment with crizotinib significantly inhibited cell proliferation of 033 T cells from day 5 onward. **d** Protein analysis of 033 T cells by western blot showed a reduction of phosphorylated MET and downstream components of MET signaling pathway, such as STAT3 and ERK1/2, following the treatment with MET inhibitor, crizotinib

In this study, we validated the applicability of lung cancer PDX models in preclinical screening of the targeted anticancer therapeutics, using five samples, including two tumor tissue samples (033 T, 694 T as negative control), two lung cancer cell lines (H1975 (*EGFR* mutation; L858R/T790 M), H2228 (*EML4-ALK*)), and a normal lung tissue sample. The IC_50_ values for 17 targeted agents were obtained using primary cultured cells in sphere culture conditions, in vitro (Additional file 3: Table S1). The drug library was composed of the targeted agents included in the clinical guidelines or currently in the clinical trials for NSCLC treatment [17]. According to the previously reported results [18], cells are considered resistant to an agent when the IC_50_ of that agent is greater than 1000 nmol/L. The proliferation of 033 T cells was not affected by EGFR inhibitors, such as erlotinib (IC_50_ = 836 nmol/L), lapatinib (IC_50_ = 1562 nmol/L) and afatinib (IC_50_ = 971 nmol/L) (Additional file 3: Table S1). However, gefitinib (IC_50_ = 382 nmol/L) and neratinib (IC_50_ = 133 nmol/L) showed marked inhibitory effects on 033 T cell proliferation, compared with the normal lung cells (Fig. 3a and Additional file 3: Table S1). NSCLC patients with *HER2* amplifications have been shown to respond to EGFR-targeted agents, including gefitinib [19]. Additionally, canertinib (IC_50_ = 80 nmol/L), a pan-HER-targeted agent, was demonstrated to be effective against 033 T PDX cells, due to *HER2* amplification in these cells (Fig. 3a). The FGFR, PDGFR, VEGFR, BRAF, CDK and Smoothened inhibitors showed little effects on 033 T PDX cell proliferation (Additional file 3: Table S1), while dual ALK/MET and PI3K inhibitors (crizotinib and buparlisib) showed low IC_50_ values (691 and 507 nmol/L, respectively). Treatment with crizotinib, MET-targeted agent, showed inhibitory effects on in vitro sphere-forming capacity and proliferation (Fig. 3b and c), by completely abolishing the phosphorylation of MET, STAT3, and ERK1/2, a downstream signaling component of MET, but not AKT phosphorylation (Fig. 3d). These results indicate that MET and HER2 signaling pathways are important for 033 T PDX cells survival and proliferation.

## Discussion

**Fig. 4:**
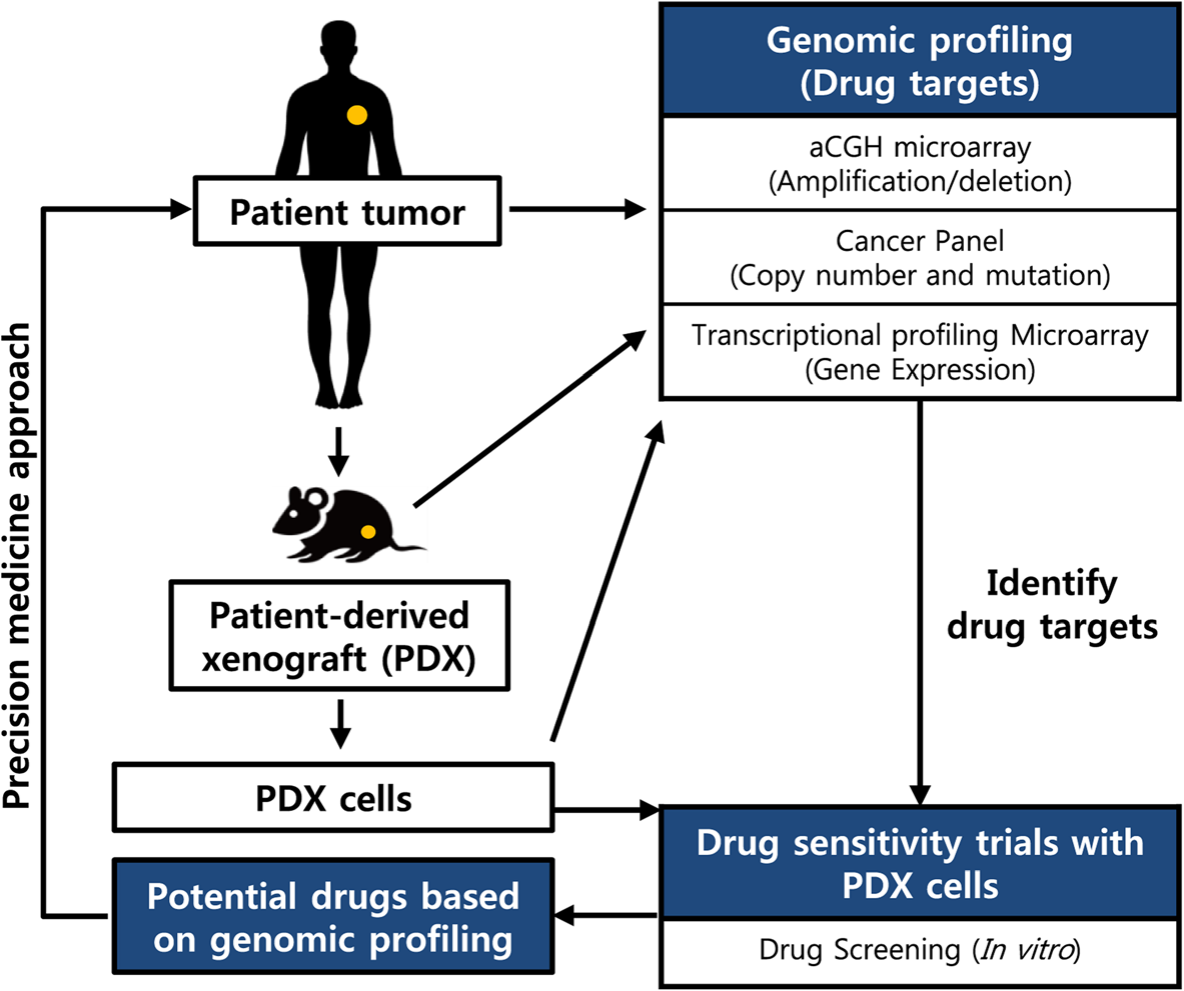
Precision medicine approach. A strategy for the application of the individualized medicine that integrates genomic profiling of a patient tumor sample, with patient-derived xenograft (PDX) and PDX cell testings, is depicted here. The genomic profiling of patient tumor samples may lead to the identification of many potential therapeutic targets. The PDX cell model can be used to test these potential drug targets, in order to determine potential therapeutic regimens for any given patient

Targeted therapy for NSCLC has developed significantly with histologic subtype and molecular subgroups harboring critical mutations as an important factor in guiding treatment. Nevertheless, the appropriate preclinical models for studies remain to be defined. We analyzed mutations, copy number variations, and protein expression using NGS-based targeted deep sequencing analysis that combines diagnostic interpretation with therapeutic suggestions for patients with cancer, as well as aCGH, and microarray with Patient tumor samples, PDXs, and PDX cells. Successful PDX growth in at least one mouse was observed for patient-derived tumors implanted subcutaneously, and PDXs were able to grow sufficiently for the transfer into other mice. The transplantable PDXs were maintained for 6 months. The successful PDX cells were isolated from the transplantable PDXs and they were maintained following two passages. The genomic profiling of the patient tumors, PDXs, and PDX cells was used to identify drug targets, and to screen PDX cells for drug sensitivity in order to select the most effective treatment candidates for the given patient (Fig. 4). In combination with several genetic profilings, the PDX models and Co-clinical trial concepts have the potential to approach the drug development in the field of precision medicine.

We demonstrated that our PDXs and PDX cell models closely recapitulated NSCLC patient tumors histopathologically and genomically by molecular biomarkers, copy number variation. Moreover, we had an experience with developing a heterotopic CC PDX model that is implanted in the subrenal capsule space to do preclinical drug efficacy studies [20]. The RTK/RAS/RAF pathway is frequently mutated in NSCLC and TKIs, which target activated protein tyrosine kinases, have demonstrated excellent therapeutic efficacy in patients with these molecular alterations [21]. Cancer-associated mutations in *KRAS* (10–30%), *EGFR* (10%) and *BRAF* (3%) in NSCLC patients are common [22]. The Cancer Genome Atlas classified NSCLCs harboring a known RTK/RAS/RAF pathway-activating somatic event as oncogene-positive samples, and found previously unrecognized genomic events, unique focal *MET* and *HER2* amplifications that might activate this critical pathway, in 38% of oncogene-negative samples, without RTK/RAS/RAF oncogene alteration and amplifications in *MET* were mutually exclusive with those in *HER2* [3]. However, we found that the tumor sample of 033 T patient diagnosed as poorly differentiated metastatic lung adenocarcinoma, PDXs, and PDX cells exhibited focal *MET* and *HER2* co-amplification and *CDKN2A* deletion without a known RTK/RAS/RAF activating mutation (Fig. 1c). Moreover, The Cancer Genome Atlas showed that co-amplification of *MET* and *HER2* was detected in 1 of the 494 lung adenocarcinoma samples (Fig. 2). 40% to 50% of patients with *MET*-amplified esophagogastric cancer harbor co-amplification of *HER2* and/or *EGFR* concurrently in the tumor cells, which can drive de novo resistance [23]. The Cancer Panel showed that *HER2* amplification was detected and progressively enriched in PDXs, and PDX cells, but not in the patient tumors, suggesting that a *HER2*-amplified clone proliferated in the PDXs and PDX cells. Another interesting point is the transition of cytoplasmic HER2 expression of the patient tumors to membranous expression of the PDX tumors. Previously, we reported that HER2 expression transitions from nuclear and cytoplasmic to membranous expression with the serial tumor passages in cervical cancer PDXs [20]. We hypothesize that the cytoplasmic HER2 protein can migrate to the cell membrane under the influence of the unfolded protein response [24], or that a minor clone that expresses membranous HER2 becomes predominate with PDX serial passages. All tumor cells from patient tumor samples and PDXs showed strong MET membranous positivity and MET showed the highest levels of expression in the PDXs (>95%; Fig. 1f, lower panel), suggesting that these changes are consistent with a selection of more aggressive tumor features in the PDXs compared with the patient tumors. Such PDX models have also been demonstrated by others to faithfully maintain histology, gene expression patterns and genomic features in NSCLC, including *MET* and *HER2*-amplified tumors. We suggest here that PDX cells could be used to investigate MET and HER2-targeted therapeutics. These models have also been shown to be effective the development of preclinical therapeutic studies for drug discovery and screening.

We tested whether the drug response profiles of PDX cells to targeted agents were concordant with the actual genomic profilings in our case. Genomically characterized ex vivo PDX cells can be used as a preclinical model to demonstrate antitumor effects of targeted agents [17]. In our study, we demonstrated that 033 T PDX cells were sensitive to EGFR TKIs and MET inhibitors in vitro. Therefore, ex vivo PDX cells maybe provide the promising solution for the investigation of precision medicine with high-throughput drug screening and this integrated analysis of genomic profilings and PDX cells sensitivity may more accurately predict clinical outcomes. However, there are several limitations to our study. Ex vivo PDX cell models do not reflect all aspects of the tumor environment, such as the presence of blood vessels, immune cells, fibroblasts, bone marrow-derived inflammatory cells, lymphocytes, and the extracellular matrix (ECM), and may not reflect the clinical treatments in patients who are treated with the targeted drug. In order to overcome this limitation, we are currently exchanging the traditional three-dimensional sphere culture method with serum-free medium for three-dimensional PDX cell culture models with matrix due to improved cell–cell interactions, cell–ECM interactions, and cell populations and structures that resemble in vivo architecture. Since 033 T PDX cells had previously been cryopreserved and could not be cultured due to limitation of primary culture, we could not investigate whether the combination of crizotinib and EGFR TKIs demonstrates better therapeutic effects against 033 T PDX cells than that with monotherapy.

## Conclusion

In this study, we demonstrate that histopathologically and genomically homologous lung cancer PDX cells models could be a valuable preclinical platform for further development of precision medicine approaches to treat lung cancer patients. Additionally, in vitro preclinical models may provide additional information predicting therapeutic response and suggest the appropriate approach for MET and HER2 treatment in MET and HER2-overexpressed lung cancer without RTK/RAS/RAF oncogene alterations.

## Additional files

Additional file 1: Figure S1. Array comparative genomic hybridization (aCGH) of patient tumors and corresponding patient-derived xenograft. The recurrence of copy number alteration is plotted on the y-axis, and each probe is aligned along the x-axis in chromosome order. Note the similarity between the genomic profiles of the patient tumors and PDX counterparts. (TIFF 208 kb)

Additional file 2: Figure S2. Retention of mRNA expression in PDXs. Results of heat maps for several potential targets and other tumor biomarkers. Higher protein expression is illustrated in red, and lower expression in green. Expression levels of most analyzed proteins in PDX were retained by the PDX cells. NC; patient 694 T samples, used as negative controls, did not show *MET* and *HER2* amplification. (TIFF 167 kb)

Additional file 3: Table S1. Table of IC50 values for various agents on a selection of cells lines. The IC50 values for 17 targeted agents were obtained using primary cultured cells in sphere culture conditions, in vitro 2. (TIFF 315 kb)

## Abbreviations

aCGH: array-comparative genomic hybridization
ALK: anaplastic lymphoma kinase
EGFR: epidermal growth factor receptor
H&E: Hematoxylin and eosin
HER2: human epidermal growth factor receptor 2
IHC: immunohistochemistry
NGS: next generation sequencing
NSCLC: Non-small-cell lung cancer
PDX: Patient-derived xenograft
PI3K: phosphatidylinositol 3-kinase
qPCR: quantitative polymerase chain reaction
RTK: receptor tyrosine kinase
STR: short tandem repeat
TKI: tyrosine kinase inhibitor
UPR: unfolded protein response.
