# Supplementary figures and images for: Precision medicine approaches to lung adenocarcinoma with concomitant MET and HER2 amplification
Source: BMC Cancer. 2017 Aug 10;17:535. doi: 10.1186/s12885-017-3525-9 (PMC5557466; doi:10.1186/s12885-017-3525-9)

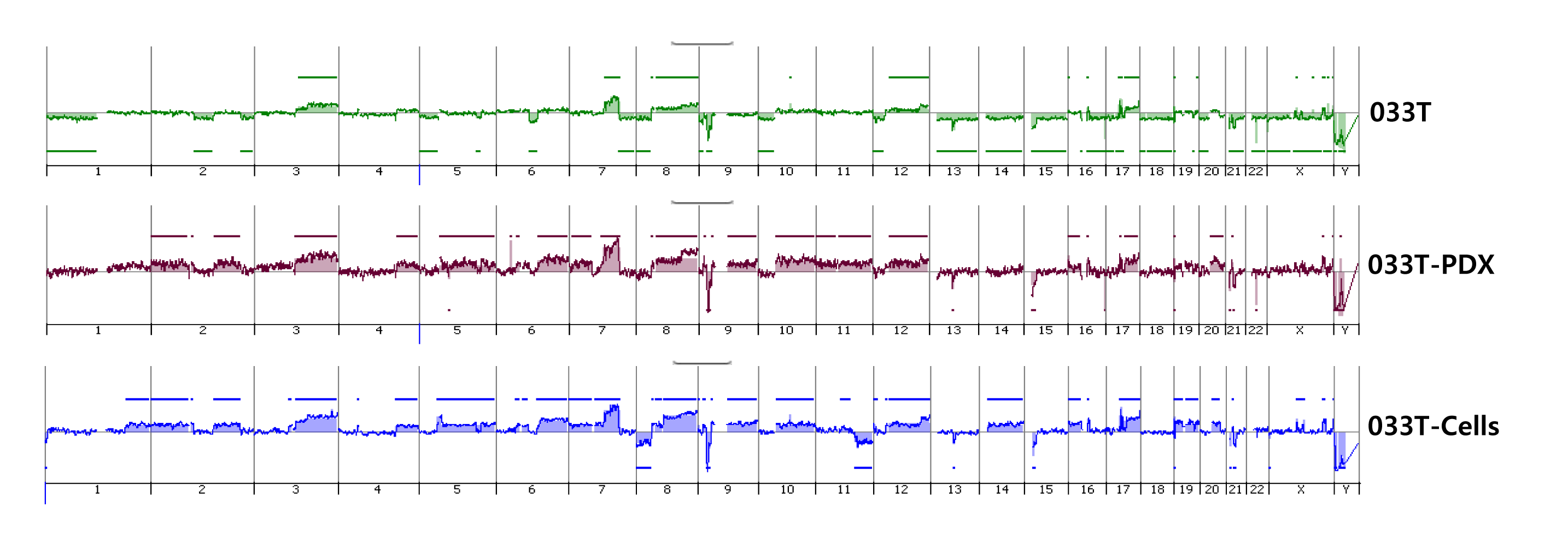

Supplement: Supplementary file 1 — Array comparative genomic hybridization (aCGH) of patient tumors and corresponding patient-derived xenograft. The recurrence of copy number alteration is plotted on the y-axis, and each probe is aligned along the x-axis in chromosome order. Note the similarity between the genomic profiles of the patient tumors and PDX counterparts. (TIFF 208 kb) [file 12885_2017_3525_MOESM1_ESM.tif]

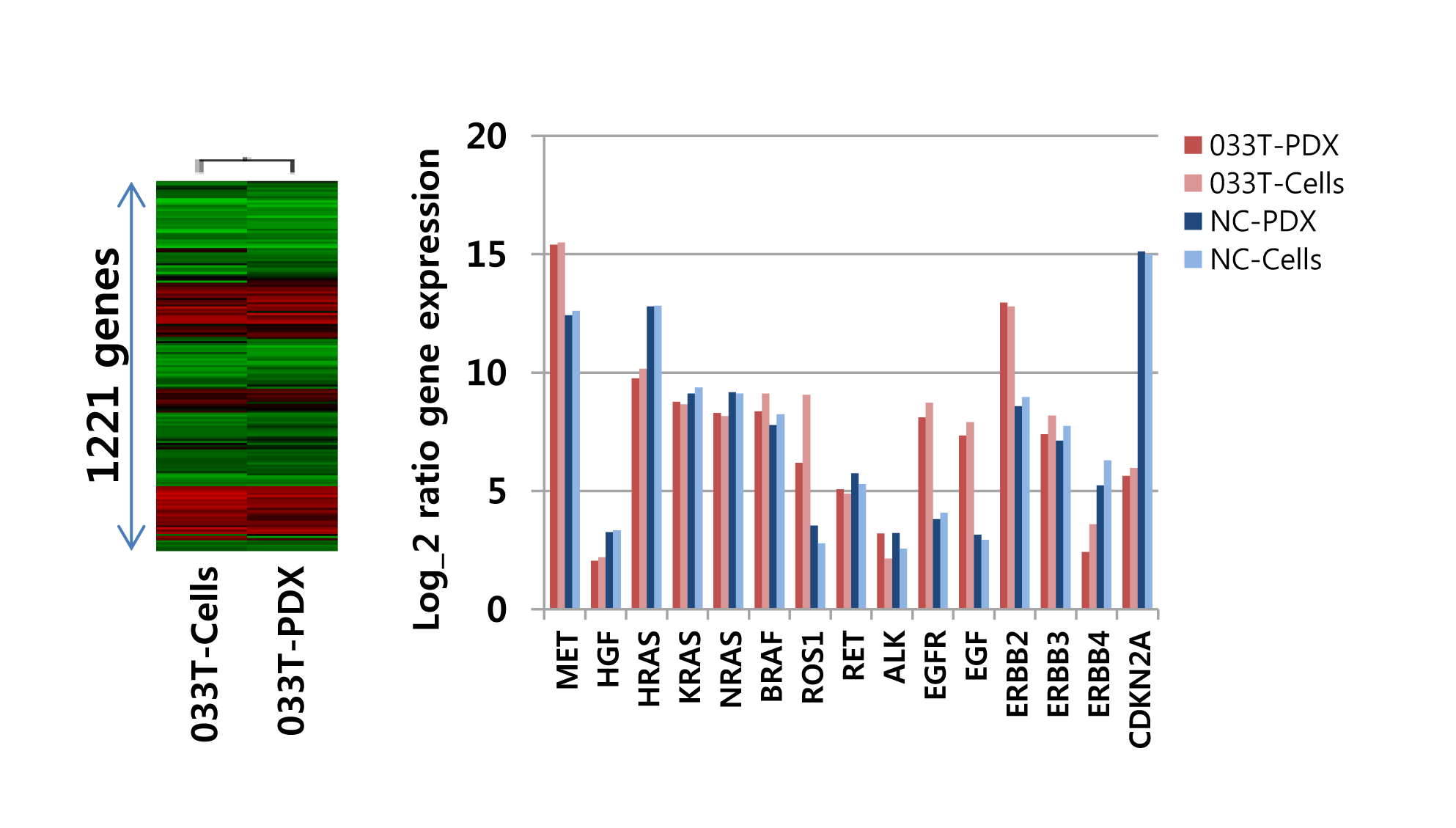

Supplement: Supplementary file 2 — Retention of mRNA expression in PDXs. Results of heat maps for several potential targets and other tumor biomarkers. Higher protein expression is illustrated in red, and lower expression in green. Expression levels of most analyzed proteins in PDX were retained by the PDX cells. NC; patient 694 T samples, used as negative controls, did not show MET and HER2 amplification. (TIFF 167 kb) [file 12885_2017_3525_MOESM2_ESM.tif]

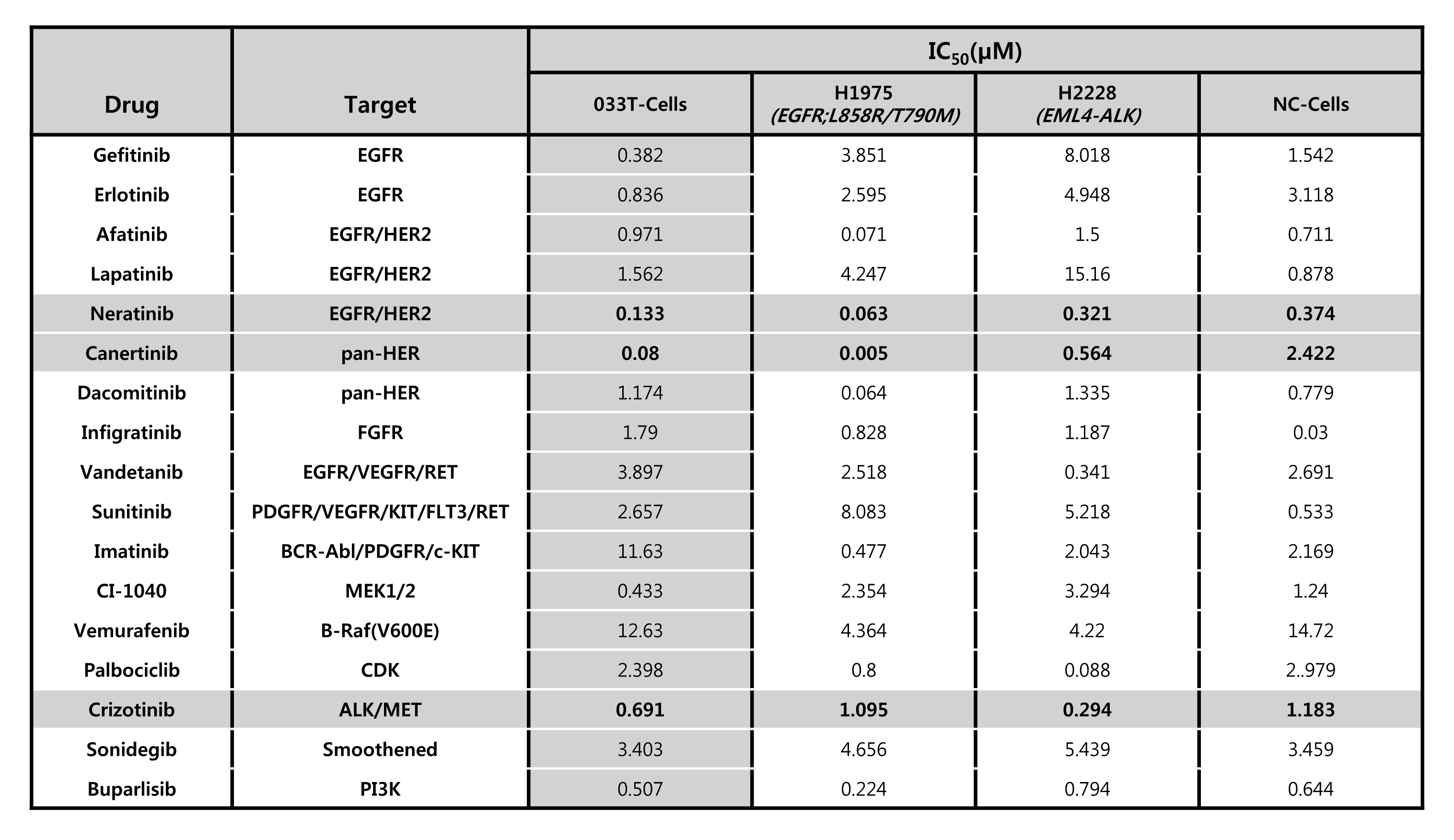

Supplement: Supplementary file 3 — Table of IC50 values for various agents on a selection of cells lines. The IC50 values for 17 targeted agents were obtained using primary cultured cells in sphere culture conditions, in vitro 2. (TIFF 315 kb) [file 12885_2017_3525_MOESM3_ESM.tif]
